# A microfluidic device for measuring cell migration towards substrate-bound and soluble chemokine gradients

**DOI:** 10.1038/srep36440

**Published:** 2016-11-07

**Authors:** Jan Schwarz, Veronika Bierbaum, Jack Merrin, Tino Frank, Robert Hauschild, Tobias Bollenbach, Savaş Tay, Michael Sixt, Matthias Mehling

**Affiliations:** 1 Institute of Science and Technology Austria (IST Austria), 3400, Klosterneuburg, Austria; 2 Department of Biosystems Science and Engineering, ETH Zürich, 4058, Basel, Switzerland; 3 Institute for Molecular Engineering, The University of Chicago, Chicago, IL, 60637, USA; 4 Department of Biomedicine and Neurology Department, University Hospital Basel, 4031, Basel, Switzerland

## Abstract

Cellular locomotion is a central hallmark of eukaryotic life. It is governed by cell-extrinsic molecular factors, which can either emerge in the soluble phase or as immobilized, often adhesive ligands. To encode for direction, every cue must be present as a spatial or temporal gradient. Here, we developed a microfluidic chamber that allows measurement of cell migration in combined response to surface immobilized and soluble molecular gradients. As a proof of principle we study the response of dendritic cells to their major guidance cues, chemokines. The majority of data on chemokine gradient sensing is based on *in vitro* studies employing soluble gradients. Despite evidence suggesting that *in vivo* chemokines are often immobilized to sugar residues, limited information is available how cells respond to immobilized chemokines. We tracked migration of dendritic cells towards immobilized gradients of the chemokine CCL21 and varying superimposed soluble gradients of CCL19. Differential migratory patterns illustrate the potential of our setup to quantitatively study the competitive response to both types of gradients. Beyond chemokines our approach is broadly applicable to alternative systems of chemo- and haptotaxis such as cells migrating along gradients of adhesion receptor ligands vs. any soluble cue.

The ability of cells to migrate is fundamental to many physiological processes, such as embryogenesis, regeneration, tissue repair and protective immunity^1^. Cell migration is mainly governed by adhesion of cells to substrates (other cells or connective tissue) and by extracellular signalling molecules acting as motogenic stimuli or directional guidance cues^2^. The specific impact of these factors differs considerably between cell types. While mesenchymal and epithelial cells are dominated by adhesive interactions the amoeboid crawling of leukocytes is largely controlled by guidance cues of the chemokine family^3^,^4^. The prevailing paradigm of chemokine function is that spatial diffusion-based gradients of chemokines induce polarization and directed migration of the responding cells towards the chemokine source^5^. However, the scarce information available for *in vivo* chemokine gradients suggests that the situation is often more complex and that chemokines are unlikely to distribute by free diffusion only. Like most growth factors chemokines bind to different degrees to cell surface or connective tissue glycosaminoglycans^6^,^7^,^8^. Such interactions restrict chemokine distribution and thereby can shape gradients. For chemokines binding with high affinity to sugar residues, immobilization can even lead to the formation of stable solid phase gradients, which induce a variant of haptotaxis^9^. Although it is conceivable that cells can equally respond to gradients of soluble and/or immobilized chemokines, almost all cell biological information available about-gradient sensing is based on *in vitro* studies employing soluble gradients.

The best understood example for the significance of immobilized vs. soluble chemokine gradients is the trafficking of dendritic cells (DCs). After having captured antigen in non-lymphoid tissues, DCs migrate along immobilized gradients of the high affinity sugar-binding chemokine (C-C motif) ligand21 (CCL21) towards lymphatic vessels, from where they are flushed into the sinus of lymph nodes. Once in the lymph node, the cells experience a second chemokine, (C-C motif) ligand19 (CCL19), which interacts with the same receptor (C-C chemokine receptor 7, CCR7) but interacts only weakly with sugars. It has been shown *in vitro* that the directionality of DCs migrating on homogenously immobilized CCL21 can be biased by gradients of soluble CCL19^10^. When exposed to competing soluble gradients of CCL19 and CCL21, DCs displayed higher sensitivity towards CCL19^11^. In contrast, if CCL21 diffusion was influenced by unspecific binding to charged extracellular matrix components, CCL21 induced directionality prevailed when opposed by a soluble CCL19 gradient^12^. How DCs respond to immobilized and co-existing immobilized and soluble chemokine gradients remains elusive.

Here we developed an *in vitro* setup to study the significance and interaction of co-existing bound and soluble chemokine gradients for directed cell migration. To this end we engineered a microfluidic device to generate diffusion-based chemokine gradients, which allows simultaneous surface-immobilization of arbitrarily graded chemokine patterns. We used DCs as a model to track migration in response to soluble and immobilized chemokine on a single cell level in real time.

## Results and Discussion

### Microfluidic system to probe chemotactic and haptotactic migration at the single cell level

**Figure 1:**
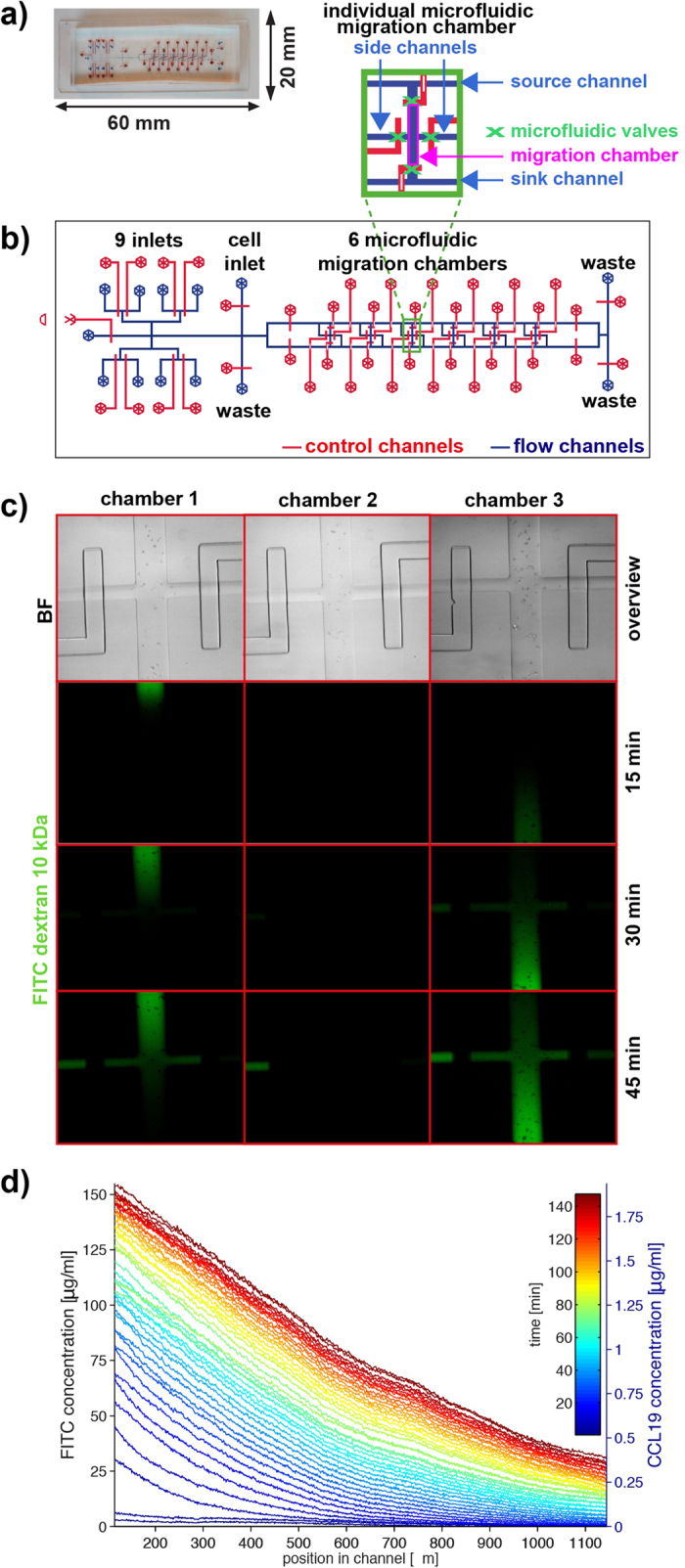
Overview of the set up and functionality of the microfluidic migration device. (**a**) Photograph of the device with flow channels filled with blue liquid and control channels filled with red liquid. (**b**) Schematic overview of the geometry of the entire device and an individual migration chamber (inset). Flow channels are shown in blue, and control channels are shown in red. (**c**) Overview of three migration chambers loaded with bone marrow derived dendritic cells (upper row) and dynamics of formation of opposing diffusion-based gradients visualized with FITC-dextran (10 kDa) in chambers one and three. In chamber two, cell culture medium is exchanged as a control. (**d**) Diffusion profiles averaged over the centered longitudinal section of the chamber as a function of the location in the chamber for different times up to 150 min, with the intensity mapped to the concentration of FITC dextran (10 kDa) (left axis) and CCL19 (right axis). Time is colour-coded from blue (short times) to red (long times), with each line separated by 3.5 min.

To quantitatively track immune cell migration in simultaneous response to chemotactic and haptotactic gradients we developed a microfluidic device that allows (i) patterning of bound chemokine gradients, (ii) precise positioning of immune cells on these haptotactic gradients and (iii) the generation of diffusion-based (flow-free) soluble chemokine gradients superimposed on haptotactic gradients in small microfluidic migration chambers. Specifically, the two-layer PDMS microfluidic device (overview in Fig. 1a) consists of 9 inlets for reagents and media, 1 cell loading inlet, 3 waste outlets and 6 migration chambers (Fig. 1b). The core component of this microfluidic device are these 6 migration chambers (l = 1100 μm, w = 200 μm, h_max_ = 28 μm) containing one side port at the middle of the long ends of the chamber while the ports at the two short ends of the chamber are connected to supporting sink and source channels (Fig. 1b). For controlled flow of fluids and cells all ports are equipped with independently controllable PDMS membrane valves. Support channels connect the reagent inlets with the migration chambers and the outlets. Cells can be loaded via the ports at the short ends resulting in a distribution along the chamber as shown in Fig. 1c. Alternatively, cells can be loaded via the ports at the long ends resulting in a localized distribution in the center of the chamber (Fig. 1c). The migration chambers are coated with fibronectin as it acts as a ligand for β_2_integrins which are expressed by DCs, induce DC adhesion and therefore allow DC migration on a 2D surface^11^. Alternatively, microfluidic devices can be coated with other extracellular matrix proteins such as laminin^13^ and our platform might therefore serve for assessing the impact of other extracellular matrix components on immune cell or cancer cell migration.

As described earlier^14^,^15^, the flow of different molecules (e.g. chemokines) through the supporting source and sink channels and coordinated opening of the respective ports to individual chambers after having stopped fluid flow allows the generation of flow-free diffusion-based chemokine gradients in which the steepness, mean concentration and duration can be independently controlled. For example, spatially opposing gradients can be generated in parallel (Fig. 1c, chamber 1 vs. chamber 3) and the polarity or ligand type of the gradients can be switched when needed. As the generation of chemotactic gradients in our device is based on diffusion for mass transport and not fluid flow, migration characteristics of individual cells can be quantified without physical disturbances of cells. Taken together, our microfluidic migration device allows tracking of individual cells with high spatial and temporal resolution.

For our CCL19 migration experiments, a continuously rising linear gradient was induced, reaching a CCL19-concentration of approximately 1.8 μg/mL at its maximum after 2.5 hrs (Fig. 1d). Our PDMS-based microfluidic system with closed channels requires bonding of one channel-sustaining PDMS part to either glass or a second PDMS layer^16^. This bonding involves activation and heating steps, which preclude protein deposition before chamber assembly. We developed a protocol allowing protein patterning on ready-assembled chips. For protein patterning, we employed a photo-patterning technique to covalently surface-deposit fluorescently tagged molecules in arbitrary shape and, most importantly, allowing for graded intensity distributions^17^,^18^.

**Figure 2:**
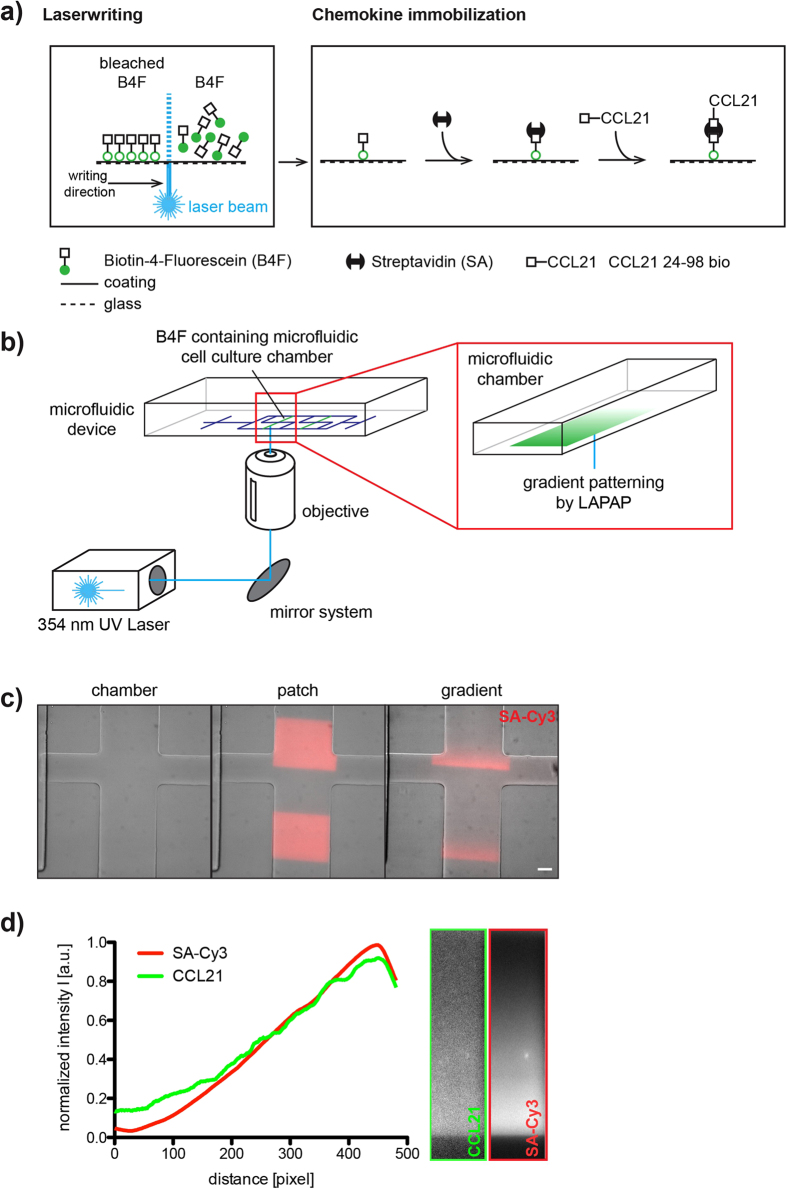
Manufacturing of haptotactic chemokine gradients in a microfluidic migration chamber. (**a**) Schematic of Laser Assisted Protein Adsorption by Photobleaching (LAPAP) of biotin-4-fluorescein (B4F, left panel) and the chemokine immobilization protocol (right panel); adapted from Schwarz & Sixt, 2016^35^. (**b**) Schematic of laser writing into a microfluidic migration chamber. PDMS block with microfluidic chip (left panel). Microfluidic migration chamber with B4F gradient (enlarged region). (**c**) SA-Cy3 staining of different laser written B4F patterns in microfluidic migration chambers overlaid with the respective bright field image of the chamber. From left to right: chamber without pattern (‘chamber’), chamber with two printed patches (‘patch’) and a chamber with two gradients (‘gradient’) as used in the migration experiments (Figs 3 and 4). Scale bar represents 100 μm. (**d**) Immunostaining of a linear CCL21 gradient printed in a migration chamber. SA-Cy3 image (red line and right image) and anti-CCL21/anti-goat AF 488 image (green line and left image). Fluorescence intensities were normalized to the respective maximum.

We patterned biotinylated fluorescein (B4F) using a focused and movable 355 nm ultra-violet laser (Fig. 2a/b). While laser positioning enables the generation of arbitrary B4F patterns, regulation of intensity and dwell time additionally allows for quantitative control of local deposition with diffraction-limited resolution. Following exposure, unbound B4F was washed out and chambers were filled with streptavidin (SA), which, upon binding to B4F, serves as an adapter for biotin-coupled reagents. Next, we loaded the chambers with the chemokine CCL21 carrying a c-terminal PEG-biotin tag (CCL21 24-98 bio). We used this truncated version of CCL21 to avoid unspecific background binding mediated by the basic c-terminal extension of full-length CCL21^19^. After washout, this yielded a surface bound CCL21 pattern, which corresponded to the initial laser pattern. The use of fluorescently labelled SA such as SA-Cy3 allowed visualization of printed patterns (Fig. 2c). Importantly, the SA-Cy3 pattern correlated closely with the amount of biotinylated CCL21 bound to SA as shown with anti-CCL21 antibody staining (Fig. 2d).

Taken together, we reconfirm previous reports of the capability to generate highly controllable diffusion-based chemokine gradients in microfluidic devices^14^,^15^. We then integrated the controlled immobilization of chemokines in this microfluidic device. The chemokine immobilization procedure involves several binding and washing steps, which are usually executed manually on a dish or cover slip. Our device not only allows patterning within the cell culture chamber but also enables automatization of all binding and washing steps because it features 10 inlets for different solutions. This accelerates the procedure of protein patterning substantially.

### Immobilized CCL21 gradients induce DC haptotaxis in a microfluidic migration chamber

**Figure 3:**
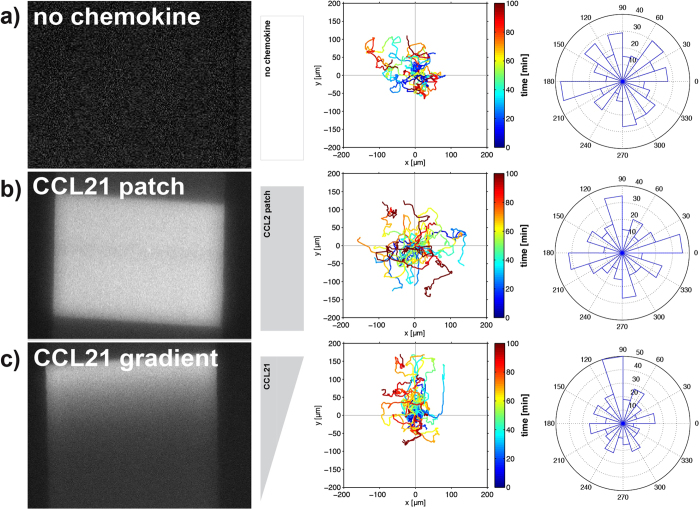
DCs migrating on immobilized CCL21 24-98 bio. (**a**) DCs migrating in a microfluidic channel treated with SA-Cy3 and CCL21 24-98 bio without B4F laser-writing. (**b**) DCs migrating on a SA-Cy3 stained CCL21 24-98 bio patch printed in a microfluidic channel. (**c**) DCs migrating on a SA-Cy3 stained CCL21 24-98 bio gradient printed in a microfluidic channel. Left panels: Representative images of the SA-Cy3 stained patterns. Middle panel: Trajectories of all cells in the respective field of view, with trajectories plotted to a common starting point [dimensions in μm; time is colour-coded]. Gradient direction and pattern shape are indicated in grey. Right panel: Rose plot visualizing the distribution of angles of all cell tracks in an angular sector field with tracks split into 3 min intervals.

The defined, surface-immobilized and bioactive CCL21 patterns and gradients obtained by LAPAP (Fig. 2c) allowed us to quantify haptokinetic and haptotactic migration of DCs in our microfluidic device. Figure 3 illustrates migration of DCs in the presence and absence of immobilized CCL21. Under control conditions DCs adhere loosely to fibronectin-coating, show a round morphology with constant protrusions (Supplemental Movie S1, no chemokine) and migrate spontaneously and undirected. This is indicated by the trajectories of all cells in this section of the migration chamber when plotted on a common starting point (Fig. 3a; middle panel) and the distribution of directionalities as illustrated by the rose plot (Fig. 3a; right panel). In contrast to this, DCs exposed to a patch with a homogenous concentration of CCL21 start to adhere more tightly to the immobilized chemokine (Supplemental Movie S1, CCL21 patch) while migrating more efficiently but undirected (Supplemental Figure S4, CCL21 patch). This demonstrates the haptokinetic effect of evenly immobilized CCL21 (Fig. 3b). While exposure to a gradient of immobilized CCL21 also resulted in more pronounced adherence of DCs to the substrate (Supplemental Movie S1, CCL21 gradient), this additionally induced directed migration of the cells towards higher concentrations of the gradient (Fig. 3c). This migration pattern demonstrates the haptotactic effect of CCL21 when immobilized as a gradient. Taken together, we show that CCL21 immobilized in our microfluidic device impacts on migration of DCs. Specifically, CCL21 induces chemokinesis when immobilized as a patch or chemotaxis when immobilized as a gradient.

### CCL19 gradients induce DC chemotaxis in a microfluidic migration chamber

**Figure 4:**
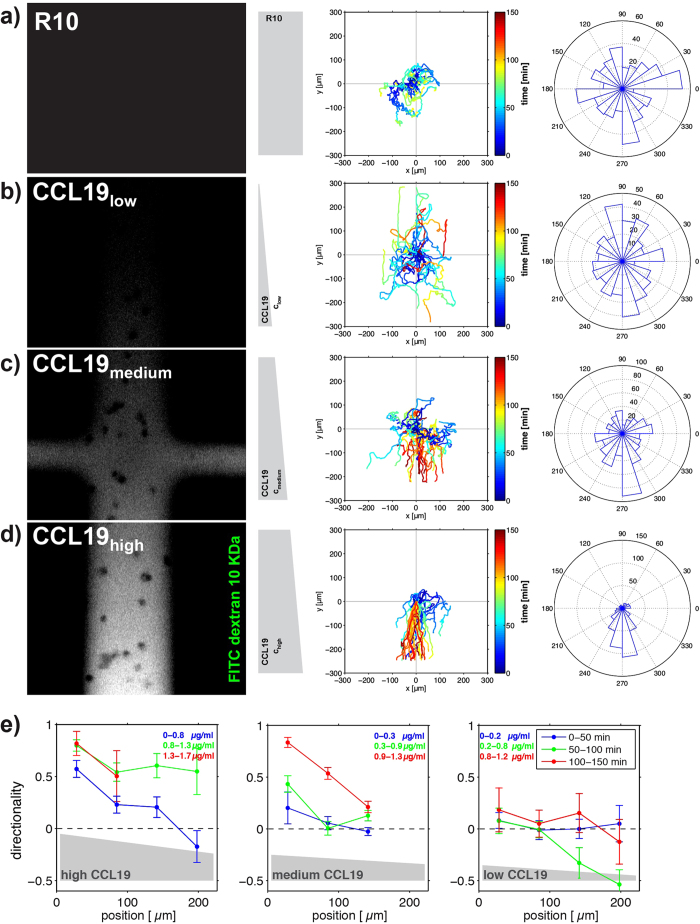
CCL19 gradients induce DC chemotaxis in a microfluidic migration chamber. (**a**) DCs migrating in a diffusion based gradient of cell culture medium (R10). (**b–d**) DCs migrating in a diffusion based CCL19/FITC dextran 10 kDa gradient. (**e)** Directionalities as a function of the position in the microfluidic assay for low (left panel) intermediate (middle panel) and high (right panel) average concentrations of CCL19. The zero position corresponds to the lower edge of the field of view in (**b–d**), where the respective concentration is maximal. The directionalities are shown for short, intermediate, and long times, shown in blue, green, and red. The concentration range covered during each period of time is indicated in the respective colour. (**b**) and (**e**). 1) Lower third of the migration chamber; low CCL19 concentration regime. (**c**) and (**e**). 2) Middle third of the migration chamber; medium CCL19 concentration regime. (**d**) and (**e**). 3) Upper third of the migration chamber; high CCL19 concentration regime. Left panel: Representative images of the CCL19/FITC dextran 10 kDa and the cell culture media (R10) gradient at t = 60 min. Middle panel: Corresponding cell track analysis of all tracks aligned to the origin [dimensions in μm; time is colour-coded]. Gradient direction and pattern shape are indicated in grey. Right panel: Rose plot visualizing the distribution of angles of all tracks in an angular sector field with tracks split into 3 min intervals.

We have previously shown that chemotaxis can be induced in T cells by exposure to a soluble gradient of the chemokine CXCL12 generated in a microfluidic migration device^14^. To recapitulate this finding for other immune cells, we exposed DCs to a continuously rising gradient of CCL19 or control conditions in migration chambers of our microfluidic device (Fig. 4). Specifically, we loaded the DCs via the ports at the short ends of the chamber resulting in a distribution of the cells along the chamber. Cells adhered loosely to the fibronectin-coated PDMS surface (Supplemental Movie S2) with some cells starting to migrate randomly within approximately 30 min (Supplemental Movie S2, 1800 s). Following attachment, we exposed the cells to fresh cell-culture medium diffusing into the chamber from both short ends as a control without generating a CCL19-gradient (Fig. 4a). This resulted in undirected migration of the cells (Fig. 4a). In parallel we exposed DCs in another migration chamber to a diffusion-based gradient of CCL19 by sequentially refilling the channels at the top and the bottom of the microfluidic migration chamber with fresh medium either containing CCL19 or cell culture medium (Fig. 4b–d). This resulted in a CCL19-gradient along the entire chamber and accordingly to exposure of DCs to low, intermediate, or high concentrations of CCL19 over time as indicated by the illustration of the gradient-formation in Fig. 1d. Specifically, cells in the upper third of the chamber are exposed earlier and ultimately also to higher concentrations of the CCL19 gradient as compared to cells in the middle and particularly the lower third of the chamber. The concentration ranges during the built-up of the CCL19-gradient for the specific sections of the chamber are indicated in Fig. 4e. Compared to control cells, exposure of DCs to low concentrations (0–1.2 μg/mL) of CCL19 resulted in faster migration (Supplemental Figure S4, CCL19 low) while overall migration remained undirected (Fig. 4b), reflecting the chemokinetic effect of the chemokine. This is in line with the with previous observations of increased migration velocity of DCs at comparable concentration ranges of CCL19^12^. The preferential distribution of tracks towards the short ends of the chamber relates to the fact that migration towards the long ends of the chamber is impeded by the rectangular geometry of the chambers. Exposure of DCs to intermediate concentrations of the CCL19-gradient (0–1.3 μg/ml) resulted in a significantly increased directionality of single cell trajectories towards higher concentrations of the chemokine (Fig. 4c). While higher CCL19-concentrations (0–1.7 μg/ml) did not influence migration velocity (Supplemental Figure S4, CCL19 high), directionality was further augmented when DCs were exposed to high concentrations of the CCL19-gradient (Fig. 4d). This is illustrated in Fig. 4e, which shows the directionality of DCs in the different section of the migration chamber during the build-up phase of the CCL19 gradient as a function of position in the respective section and time. As shown in Fig. 4a–d, the chamber was divided into 3 sections containing high, intermediate and low concentrations of the CCL19-gradient. These data show that DCs migrate in high concentrations of the chemokine gradient more directional than in intermediate concentrations while migration in low concentrations was non-directional. Taken together, these data indicate that directionality of DC migration correlated with increasing concentrations of CCL19 in the gradient. The CCL19-concentration in our gradients reached up to 1.7 μg/mL (approximately 190 nM) and therefore covered the dissociation constant of the interaction of CCR7 with CCL19 (K_D_ CCR7/CCL19: 10 nM–100 nM)^12^,^20^. Also, these concentrations have been described to sufficiently trigger downstream signalling of CCR7 following binding of CCL19 in G protein loading assays^21^ and downstream signalling assays^22^.

In summary, we show that diffusion-based chemokine gradients in microfluidic devices can induce directed migration also in DCs. The finding that directed migration in microfluidic devices can also be induced in myeloid cells expands previous reports on the induction of directed migration in T lymphocytes^14^ and emphasizes the potential of microfluidics for assessing biologically relevant properties on a single cell level.

### Migration of DCs in competing chemotactic and haptotactic chemokine gradients

**Figure 5:**
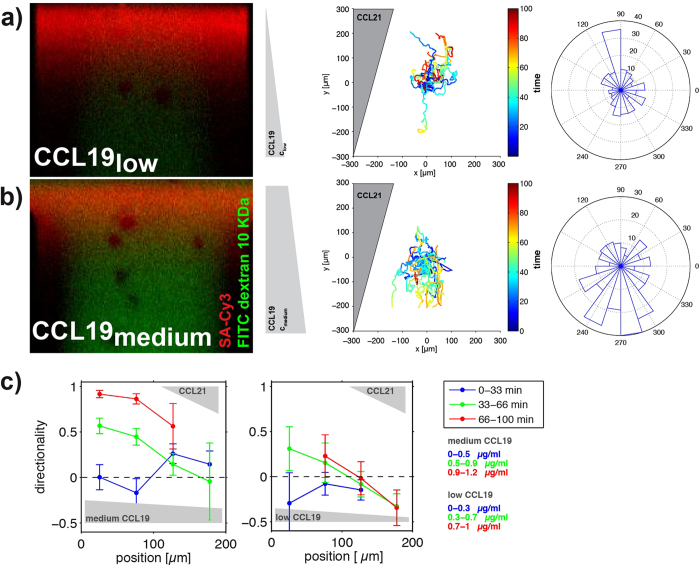
DCs migrating on opposing chemotactic CCL19 and haptotactic CCL21 gradients. (**a**) CCL19_low_/CCL21 area of the chamber. (**b**) CCL19_medium_/CCL21 area of the chamber. Left panel: Representative images of the soluble CCL19/FITC dextran 10 kDa gradient (green) after 45 min superimposed with immobilized CCL21/SA-Cy3 gradients (red) of the respective areas. Middle panel: Tracks of migrated DCs in the respective areas aligned to the origin [dimensions in μm; time is colour-coded]. Gradient directions are indicated in grey. Right panel: Rose plot visualizing the distribution of angles of all tracks in an angular sector field (3 min intervals). (**c**) Directionalities as a function of the position in the channel for intermediate and low average concentrations of CCL19 (from left to right). Zero position corresponds to the lower edge of the field of view, and the directionalities are shown in blue, green, and red for short, intermediate, and long times. As in Figure 4, the concentration ranges per time span are indicated in the corresponding colour.

After having shown that our microfluidic device allows exposing DCs to chemotactic and haptotactic guidance cues we next quantified migration of DCs simultaneously exposed to diffusion-based and immobilized chemokine gradients. Specifically, we assessed migration of DCs when exposed to competing gradients of soluble CCL19 on the one side and immobilized CCL21 on the other side. To this end CCL21-gradients were printed into the lower and middle area of the migration chamber, with a maximal concentration of ca. 160 molecules/μm^2^ (which, if related to unit volume, corresponds to a concentration of 0.24 μg/mL, see Supplemental Figure S6). After washing, DCs were positioned in the chamber including the two CCL21-gradients (Supplemental Movie S3). After 30 min of cell recovery, an opposing diffusion-based CCL19 gradient (Supplemental Figure S5) was generated as described above. By doing so, one of the CCL21-gradients was superimposed with a low-concentration CCL19-gradient (CCL19low (0–1 μg/mL)/CCL21 gradients; Fig. 5a), while the other was superimposed with a medium-concentration CCL19-gradient (CCL19medium (0–1.2 μg/mL)/CCL21 gradients; Fig. 5b). DCs positioned in CCL19low/CCL21 gradients migrated towards higher concentrations of the haptotactic CCL21-gradient (Fig. 5a). Specifically, cells migrate in a haptotactic fashion towards the higher concentrations of the CCL21-gradient at all times as indicated by the colour code (cold colours: early time-points, hot colours: later time-points) in Fig. 5a. In contrast to this, DCs positioned in CCL19medium/CCL21 gradients migrated towards higher concentrations of the chemotactic CCL19-gradient (Fig. 5b). As for the chemotactic gradient profiles, we plotted the average directionalities of DCs in the CCL19low/CCL21 and the CCL19medium/CCL21 gradients as a function of time (Fig. 5c). These data indicate that the presence of the CCL19low/CCL21gradients induced preferential migration towards the haptotactic gradient. By contrast, exposure of DCs to CCL19 medium/CCL21 gradients resulted, after build-up of the CCL19-gradient, in highly directional chemotaxis. Our findings suggest that haptotactic CCL21 gradients in the presence of a soluble CCL19-gradient induce directed migration only at low CCL19 concentrations. Increasing concentrations of the CCL19-gradient resulted in directed migration along the soluble gradient, while overriding the effect of the opposing haptotactic CCL21-gradient. Whether the weak directional response of DCs to immobilized CCL21 in the presence of a competing gradient of CCL19 relates to the comparable binding affinities of CCL19 and CCL21 to CCR7 23,12 remains unclear. Alternatively, differences in the binding sites on CCR7 might cause this result. Although the overall tertiary structure of chemokines is highly conserved, single amino acid mutation studies have shown that activation of CCR7 by CCL19 and CCL21 can be differentially.affected^24^. These data indicate that CCL19 and CCL21 mostly share their binding sites on CCR7 but require at least one different interaction for activating CCR7. Independent of the underlying molecular mechanism our setup might contribute to address questions whether and how recognition of immobilized CCL21 differs from the recognition of soluble guidance cues.

Taken together, we show that the use of microfluidics allows superimposing chemotactic on haptotactic chemokine gradients and that DCs respond differentially to these guidance cues. As the characteristics of these soluble and immobilized chemokine gradients can be precisely controlled, this approach has the potential to address fundamental questions of directional cell migration.

## Conclusion

Much of our understanding of directed cell migration is based on data from animal models. Some of the findings have been recapitulated *in vitro* by either chemotactic or haptotactic migration assays. These data have added substantially to our understanding of the mechanisms underlying guided cell migration. However, the likely possibility that both chemotactic and haptotactic gradients exist simultaneously *in vivo* was not addressed in such setups.

The setup described here allows the generation of haptotactic gradients by photo-patterning and the flow-free generation and maintenance of diffusion-based gradients. Therefore, our setup allows for the first time and in contrast to previous reports^11^,^12^ a highly precise control of combined immobilized and soluble chemokine gradients. The integration of these assays into a microfluidic device allows positioning of cells in specific regions of the respective gradients and assessing responses to a multitude of guidance cues in parallel. As a proof of concept we compared quantitatively migration characteristics of DCs on immobilized chemokine gradients with varying superimposed chemotactic gradients and found differential migratory responses. In peripheral tissues like skin CCL21 forms an immobilized gradient, guiding DCs towards lymphatic vessels. At the same time soluble CCL19 is produced mainly by cells of the hematopoietic lineage and there is indirect *in vivo* evidence that the fine-regulation of CCL19 in the skin can rate-limit DC migration^25^. Our finding that soluble CCL19 can override the immobilized CCL21-signal might suggest that CCL19 could antagonize CCL21 and serve as a local retention signal.

Hence, our microfluidic setup is suited to study processes in which co-existing immobilized and soluble gradients impact on cell migration. In addition to immobilizing chemokines, our approach qualifies also to study migration characteristics of cells in haptotactic gradients of cellular adhesion sites – e.g. along gradients of integrin-ligands and co-existing gradients of any conceivable soluble guidance cue.

An example for co-existing immobilized and soluble chemokine gradients is the mobilization of mesenchymal stem cells during tissue regeneration by soluble chemokines such as CCL19 on the one side and chemokines that bind to extracellular glycosaminoglycans such as CCL2, CXCL12 or CCL5 on the other side^26^,^27^. Also, guided cell migration presumably mediated by chemotactic and haptotactic signals are of importance in the pathogenesis of autoimmune diseases such as multiple sclerosis (MS)^28^. This assumption is underlined by the fact that potent drugs for the treatment of MS target immune cell migration^29^,^30^. Taken together, the combination of chemo- and haptotactic guidance cues plays a fundamental role during various physiological processes such as protective immunity and tissue regeneration but also in the pathogenesis of diseases like cancer, metastasis and autoimmunity. In light of the capability of our microfluidic device to control co-existing chemotactic and haptotactic guidance cues and assess the migratory response of cells on a single cell level in real time our setup has the potential to significantly contribute to a better understanding of migration-related aspects of the above-mentioned processes.

## Material and Methods

### Mice

C57BL/6J mice used in this study were bred and maintained in accordance with the Austrian law for animal experiments (“Österreichisches Tierschutzgesetz”) and sacrificed at 4 to 10 weeks of age for use in experiments. Permission and all experimental protocols were approved by the Austrian federal ministry of science, research and economy (identification code: BMWF-66.018/0005-II/3b/2012). All experiments were performed in accordance with relevant guidelines and regulations.

### Chemokine

Murine CCL19 was purchased from R&D systems, USA. Biotinylated, truncated murine CCL21 (mCCL21 24–98 bio) was synthesized by ALMAC (Craigavon, UK). Chemokines were reconstituted as follows: Desiccated chemokines were reconstituted to a concentration of 25 μg/mL in PBS and stored at −20 °C. Prior to use, mCCL21 24–98 bio was diluted to a working concentration of 250 ng/mL in PBS.

DyLight 594 (Thermo Fisher Scientific) labeled mCCL21 24–98 bio was prepared following the manufacturers protocol. Briefly, 100 μg mCCL21 24–98 bio were reconstituted in 100 μL phosphate buffer containing 0.1 M Na_2_HPO_4_, 0.15 M NaCl adjusted to pH 7.2–7.5. 65 μg DyLight 594 NHS ester (Thermo Fisher Scientific) were added and the mixture was allowed to react for 1 h at room temperature. Subsequently, excessive DyLight 594 NHS ester was quenched for 1 h at room temperature by adding 1.4 mL of Tris/HCl pH 7.6. mCCL21 24–98 bio DL595 was purified using MW10 kDa spin columns (Amicon Ultra-2 Centrifugal Filter devices, Millipore) and stored at −20 °C.

### Quantification of surface immobilized CCL21 24–98 bio

Fluorescence intensities of a dilution series of mCCL21 24–98 bio DL595 (10, 2.5, 0.25 and 0.025 μg/mL) were measured in a defined volume of a 10 μm high PDMS chamber (manufactured similarly as microfluidic chips) and a standard curve was calculated (Fluorescence intensity = 3443x molecules/μm^2^ R^2^ = 0.97633). Fluorescence intensities of patches of surface immobilized mCCL21 24–98 bio DL595 were measured using the same imaging settings as for the dilution series. Immobilized mCCL21 24–98 bio DL595 concentrations were calculated from measured fluorescence intensities using the obtained standard curve.

### Design and fabrication of microfluidic chips

The microfluidic photomask design was drawn with Coreldraw X6 (Corel corporation, US) and printed on transparency at a resolution of 8 μm (JD Photo Data & Photo Tools, UK). A control and flow mold were produced by photo-lithography on a silicon wafer as described earlier with minor modifications^14^. In brief, the flow layer mold was spin-coated with hexamethyldisiloxane at 3000 rpm for 30 s and then baked at 110 °C for 1 min. Next, the wafer was spin-coated with AZ-40XT (Microchemicals, Germany) at 3000 rpm for 30 s and soft baked at 110 °C for 5 min. Photoresist was then exposed to ultra violet (UV) light for 15 min using a beam expanded 365 nm UV LED, (M365L2-C1–UV, Thorlabs GmbH, Germany). After UV exposure, the wafer was post-baked for 2 min at 110 °C. The wafer was developed in AZ- 726-MIF developer for 5–7 min, rinsed in water and was then reflowed for valve closing at 110 °C for 10 min. The 100 μm wide parabolic AZ40XT channels had a central height of 26.3 μm. The control layer silicon wafer mold was spin-coated with GM1070 SU-8 (Gersteltec, Switzerland) at 3100 rpm for 40 s to reach a final height of 25 μm. The wafer was then baked for 15 min at 65 °C and then 35 min at 95 °C. The wafer was exposed for 10 min with the UV-LED. The post exposure bake was 15 min at 65 °C, then 45 min at 95 °C. The wafer was then developed in SU-8 developer (Gersteltec, Switzerland). Finally, both flow and control wafers were non-stick functionalized with trichlorosilane for 1 hour in a vacuum desiccator. Microfluidic chips were fabricated by multi-layer polydimethylsiloxane (PDMS) soft-lithography as described previously^14^,^15^.

### Chip set-up, operation and control

The glass slide carrying the microfluidic chip was cleaned and taped on a slide holder. Control channels were connected to miniature pneumatic solenoid valves (Festo, Switzerland) that were controlled via an established control box system^31^ with a custom Matlab (MathWorks, US) graphical user interface. Optimal closing pressures of push-up PDMS membrane valves were determined individually for each chip the pressure in control channels was increased by 0.5 bar. Flow lines were connected to inlets, pressurized with 0.2–0.4 bar and the whole chip was filled with phosphate buffered saline (PBS). The cell culture chambers were incubated with human plasma fibronectin (c = 250 μg/mL, Millipore, Austria) for 1 hour while fibronectin remaining in the flow channels was flushed off the flow channels with PBS. Following incubation of cell culture chambers for 1 hour with fibronectin, the entire chip was flushed with cell culture medium for 10 min.

### Generation of stable soluble chemokine gradients

Stable diffusion-based chemokine gradients were generated and maintained as previously described by using a switching source-sink flow pattern^14^,^15^. Briefly, the channels at the short ends of the cell culture chambers were sequentially refilled with fresh R10 medium (RPMI 1640 supplemented with 10% fetal calf serum, L-Glucose and Penicillin/Streptomycin, all from Life Sciences, Austria) or with a mixture of murine CCL19 (2.5 μg/mL in R10; R&D systems USA) and FITC-dextran 10 kDa (200 μg/mL in R10; hydrodynamic radius: 2.3 nm; Sigma Aldrich, US). By doing so, a local high concentration (source) and a low concentration (sink) is established between which a chemokine gradient is built up and maintained by diffusion. We used FITC-dextran as a proxy to monitor the chemokine gradient within the chamber. The diffusion profiles of FITC-dextran 10 kDa and the chemokines CCL19 and CCL21 are expected to be very similar for similar hydrodynamic radii. These radii can be estimated empirically^32^ and read (1.7 +/− 0.4) nm for CCL19 (9 kDa) and (1.9 +/− 0.4) nm for CCL21 (12.5 kDa), which is comparable to the 2.3 nm of FITC.

### Generation of bound chemokine gradients by laser-assisted adsorption by photobleaching

For *on-chip* chemokine patterning, each chamber was washed with PBS for 10 s to remove unbound fibronectin. Next, chambers were filled with biotin-4-fluorescein (B4F, 150 μg/mL, Sigma Aldrich, US) and patterns were written using a steerable, pulsed UV laser (λ = 355 nm) Specifically, a long working distance 20x objective (Zeiss LD Plan Neo 20 × 0.4) focused the UV laser at the interface between the bottom of the microfluidic chamber and the B4F. A custom program controlled a pair of high-speed galvanometric mirrors that moved the focus spot within the chamber. The gradient pattern was specified by an image whose pixel values determined the light dose used for bleaching. Careful calibration allowed compensating for the off-center drop of numerical aperture of the objective as well as the geometric distortions arising from the imperfect imaging of the scan mirrors into the back aperture of the objective. Hence, the full field of view of the objective could be utilized for gradient writing. For each spot, the total light dose was split up into multiple laser pulses in order to average out the pulse-to-pulse power variability of the laser. The gradient was written into the bottom of the chambers one spot at a time with the scanning mirrors moving the laser focus by about half the diameter of the focus spot in order to create a continuous pattern. The low wavelength of the UV laser resulted in a high lateral resolution (~0.7 μm) and the low crosstalk to a high dynamic range (~100:1) of the gradient pattern. The writing speed was limited by the laser’s pulse frequency of 1 kHz. A full description of the hardware employed can be found in Behrndt *et al*.^33^. Following laser writing, the chamber was washed with PBS for 10 s and subsequently incubated for 20 min at room temperature with streptavidin-Cy3 (SA-Cy3, 10 μg/mL in PBS with 3% BSA, Sigma Aldrich, US). After 10 s of washing with PBS, the chamber was incubated for 30 min at 37 °C with biotinylated CCL21 (CCL21 24–98 bio; custom synthesized, 250 ng/mL in PBS, Almac, UK). Apart from loading steps, the supporting source and sink channels were kept constantly under flow with PBS (0.2 bar) to reduce unspecific adsorption of any reagent outside the cell culture chambers. Following washing for 10 s with PBS the DC suspension (10 × 10^6^ cells/mL in R10 medium) was loaded into the cell culture chambers.

The concentration of immobilized CCL21 was quantified indirectly by measuring the intensities of different concentrations of soluble CCL21 bio with molecular weight 9080 Da in a given volume of the chamber. These intensities were then matched to the amount of molecules in that volume. As intensities are seen as a projection across the height of the chamber, the intensities observed can be mapped to the number of CCL21 molecules per unit area. The maximal intensity observed for immobilized CCL21 then corresponds to (157 ± 33) molecules/μm^2^ (Supplemental Figure S6).

### Dendritic cell isolation, culture and maturation

DCs were generated from bone marrow cells extracted from femur and tibia of C57BL6 mice. In brief, bone marrow cells were collected by spinning distally capped bones in an upright position with 4500 rpm for 5 min. Next, 2 × 10^6^ bone marrow cells were cultured in 10 mL R10 medium containing 1 mL supernatant from a granulocyte-macrophage colony-stimulating factor (GM-CSF) hybridoma cell line in a non-adhesive petri dish. On day 4, 10 mL of R10 medium containing 2 mL supernatant from a GM-CSF hybridoma cell line were added. On day 7, 10 mL of cell culture medium was replaced by 10 mL of R10 medium containing 2 mL supernatant from a GM-CSF hybridoma cell line. DCs were harvested on day 8–10 of the culture and maturated overnight with lipopolysaccharide (LPS, 200 ng/mL).

### Imaging, Cell-tracking and data analysis

Cells were imaged using an automated inverted microscope (Nikon Ti, 10 × /NA 0.3 Air Plan Fluor Ph1 and 20 × /NA 0.5 Air Plan Fluor Ph1 objective; Nikon, Japan) equipped with a stage-top incubator controlling for temperature (37 °C), CO_2_-concentration (5%) and humidity (90%), a digital EMCCD camera (EMCCD C9100–02; Hamamatsu photonics, Japan) and the imaging software Nikon NIS-AR (Nikon, Japan). For evaluation of migration properties, cells were tracked in an area of 300 × 200 μm, which corresponds to the size of the chemokine pattern. Cell tracks are represented on an x-y coordinate system, with the origin of each trajectory aligned to (0,0). Each track is colour-coded for time, such that cold colours represent early and hot colours later time points. For image processing and cell tracking, Fiji^34^ and a plugin for manual tracking (“Manual Tracking”, Cordelieres 2005) were used. Images and tracking data were analyzed using Matlab 2013 (MathWorks Inc., US).

### Statistical analysis

From cell tracks, the direction of the cells was calculated through the angle **Θ** between the direction of the respective gradient and the (current) cell direction. The direction of each cell was determined every 3 min. This time interval was chosen so that most cells have moved by roughly their typical diameter. Events, without any motion, were excluded from the analysis. The overall directionality of cells is then given by <cos(**Θ**)>, where the average is over both time and location of the cells, with time spans and regions as indicated in the main text. Error bars represent the standard deviation of <cos(**Θ**)> determined with a bootstrapping method, where we resampled 200 times with the original sample size.

## Additional Information

**How to cite this article**: Schwarz, J. *et al*. A microfluidic device for measuring cell migration towards substrate-bound and soluble chemokine gradients. *Sci. Rep*. **6**, 36440; doi: 10.1038/srep36440 (2016).

**Publisher’s note**: Springer Nature remains neutral with regard to jurisdictional claims in published maps and institutional affiliations.

## Supplementary Material

Supplementary Information

Supplementary Movie S1

Supplementary Movie S2

Supplementary Movie S3

## References

[b1] Horwitz R. & Webb D. Cell migration. Current Biology 13, R756–R759 (2003). 14521851 10.1016/j.cub.2003.09.014

[b2] Ridley A. J. Cell Migration: Integrating Signals from Front to Back. Science 302, 1704–1709 (2003). 14657486 10.1126/science.1092053

[b3] Bear J. E. & Haugh J. M. Directed migration of mesenchymal cells: where signaling and the cytoskeleton meet. Current Opinion in Cell Biology 30C, 74–82 (2014). 10.1016/j.ceb.2014.06.005 PMC4177959 24999834

[b4] Thelen M. Dancing to the tune of chemokines. Nat Immunol 2, 129–134 (2001). 11175805 10.1038/84224

[b5] Roca-Cusachs P., Sunyer R. & Trepat X. Mechanical guidance of cell migration: lessons from chemotaxis. Current Opinion in Cell Biology 25, 543–549 (2013). 23726023 10.1016/j.ceb.2013.04.010

[b6] Patel D. D. . Chemokines Have Diverse Abilities to Form Solid Phase Gradients. Clinical Immunology 99, 43–52 (2001). 11286540 10.1006/clim.2000.4997

[b7] Bao X. . Endothelial Heparan Sulfate Controls Chemokine Presentation in Recruitment of Lymphocytes and Dendritic Cells to Lymph Nodes. Immunity 33, 817–829 (2010). 21093315 10.1016/j.immuni.2010.10.018 PMC2996097

[b8] Sarris M. . Inflammatory chemokines direct and restrict leukocyte migration within live tissues as glycan-bound gradients. Curr. Biol. 22, 2375–2382 (2012). 23219724 10.1016/j.cub.2012.11.018

[b9] Weber M. . Interstitial Dendritic Cell Guidance by Haptotactic Chemokine Gradients. Science 339, 328–332 (2013). 23329049 10.1126/science.1228456

[b10] Schumann K. . Immobilized Chemokine Fields and Soluble Chemokine Gradients Cooperatively Shape Migration Patterns of Dendritic Cells. Immunity 32, 703–713 (2010). 20471289 10.1016/j.immuni.2010.04.017

[b11] Ricart B. G., John B., Lee D., Hunter C. A. & Hammer D. A. Dendritic Cells Distinguish Individual Chemokine Signals through CCR7 and CXCR4. The Journal of Immunology 186, 53–61 (2010). 21106854 10.4049/jimmunol.1002358

[b12] Haessler U., Pisano M., Wu M. & Swartz M. A. Dendritic cell chemotaxis in 3D under defined chemokine gradients reveals differential response to ligands CCL21 and CCL19. Proceedings of the National Academy of Sciences of the United States of America 108, 5614–5619 (2011). 21422278 10.1073/pnas.1014920108 PMC3078419

[b13] Dertinger S. K. W., Jiang X., Li Z., Murthy V. N. & Whitesides G. M. Gradients of substrate-bound laminin orient axonal specification of neurons. Proceedings of the National Academy of Sciences of the United States of America 99, 12542–12547 (2002). 12237407 10.1073/pnas.192457199 PMC130496

[b14] Mehling M., Frank T., Albayrak C. & Tay S. Real-time tracking, retrieval and gene expression analysis of migrating human T cells. Lab Chip 15, 1276–1283 (2015). 25512266 10.1039/c4lc01038h

[b15] Frank T. & Tay S. Flow-switching allows independently programmable, extremely stable, high-throughput diffusion-based gradients. Lab Chip 13, 1273 (2013). 23386049 10.1039/c3lc41076e

[b16] Kellogg R. A., Gomez-Sjöberg R., Leyrat A. A. & Tay S. S. High-throughput microfluidic single-cell analysispipeline for studies of signaling dynamics. Nat Protoc 9, 1713–1726 (2014). 24967621 10.1038/nprot.2014.120

[b17] Holden M. A. & Cremer P. S. Light activated patterning of dye-labeled molecules on surfaces. J. Am. Chem. Soc. 125, 8074–8075 (2003). 12837056 10.1021/ja035390e

[b18] Bélisle J. M., Correia J. P., Wiseman P. W., Kennedy T. E. & Costantino S. Patterning protein concentration using laser-assisted adsorption by photobleaching, LAPAP. Lab Chip 8, 2164 (2008). 19023482 10.1039/b813897d

[b19] Hirose J. . Chondroitin sulfate B exerts its inhibitory effect on secondary lymphoid tissue chemokine (SLC) by binding to the C-terminus of SLC. Biochim Biophys Acta 1571, 219–224 (2002). 12090936 10.1016/s0304-4165(02)00232-5

[b20] Yoshida R. . Secondary lymphoid-tissue chemokine is a functional ligand for the CC chemokine receptor CCR7. Journal of Biological Chemistry 273, 7118–7122 (1998). 9507024 10.1074/jbc.273.12.7118

[b21] Kohout T. A. . Differential Desensitization, Receptor Phosphorylation, -Arrestin Recruitment, and ERK1/2 Activation by the Two Endogenous Ligands for the CC Chemokine Receptor 7. Journal of Biological Chemistry 279, 23214–23222 (2004). 15054093 10.1074/jbc.M402125200

[b22] Otero C., Groettrup M. & Legler D. F. Opposite fate of endocytosed CCR7 and its ligands: recycling versus degradation. J. Immunol. 177, 2314–2323 (2006). 16887992 10.4049/jimmunol.177.4.2314

[b23] Sullivan S. K., McGrath D. A., Grigoriadis D. E. & Bacon, K. B. Pharmacological and Signaling Analysis of Human Chemokine Receptor CCR-7 Stably Expressed in HEK- 293 Cells: High-Affinity Binding of Recombinant Ligands MIP-3β and SLC Stimulates Multiple Signaling Cascades. Biochem. Biophys. Res. Commun. 263, 685–690 (1999). 10512740 10.1006/bbrc.1999.1442

[b24] Ott T. R., Pahuja A., Nickolls S. A., Alleva D. G. & Struthers R. S. Identification of CC chemokine receptor 7 residues important for receptor activation. Journal of Biological Chemistry 279, 42383–42392 (2004). 15284247 10.1074/jbc.M401097200

[b25] Bryce S. A. . ACKR4 on Stromal Cells Scavenges CCL19 To Enable CCR7-Dependent Trafficking of APCs from Inflamed Skin to Lymph Nodes. The Journal of Immunology 196, 3341–3353 (2016). 26976955 10.4049/jimmunol.1501542

[b26] Hocking A. M. The Role of Chemokines in Mesenchymal Stem Cell Homing to Wounds. Adv Wound Care (New Rochelle) 4, 623–630 (2015). 26543676 10.1089/wound.2014.0579 PMC4620518

[b27] Fox J. M., Chamberlain G., Ashton B. A. & Middleton J. Recent advances into the understanding of mesenchymal stem cell trafficking. Br. J. Haematol. 137, 491–502 (2007). 17539772 10.1111/j.1365-2141.2007.06610.x

[b28] Holman D. W., Klein R. S. & Ransohoff R. M. The blood-brain barrier, chemokines and multiple sclerosis. Biochimica Et Biophysica Acta-Molecular Basis of Disease 1812, 220–230 (2011). 10.1016/j.bbadis.2010.07.019 PMC3005102 20692338

[b29] Mehling M. . Antigen-specific adaptive immune responses in fingolimod-treated multiple sclerosis patients. Ann. Neurol. 69, 408–413 (2011). 21387383 10.1002/ana.22352

[b30] Derfuss T., Kuhle J., Lindberg R. & Kappos L. Natalizumab therapy for multiple sclerosis. Semin Neurol 33, 26–36 (2013). 23709210 10.1055/s-0033-1343793

[b31] Gomez-Sjöberg R., Leyrat A. A., Pirone D. M., Chen C. S. & Quake S. R. Versatile, fully automated, microfluidic cell culture system. Anal Chem 79, 8557–8563 (2007). 17953452 10.1021/ac071311w

[b32] Wilkins D. K. . Hydrodynamic radii of native and denatured proteins measured by pulse field gradient NMR techniques. Biochemistry 38, 16424–16431 (1999). 10600103 10.1021/bi991765q

[b33] Behrndt M. . Forces driving epithelial spreading in zebrafish gastrulation. Science 338, 257–260 (2012). 23066079 10.1126/science.1224143

[b34] Schindelin J. . Fiji: an open-source platform for biological-image analysis. Nat Meth 9, 676–682 (2012). 10.1038/nmeth.2019 PMC3855844 22743772

[b35] Schwarz J. & Sixt M. Quantitative Analysis of Dendritic Cell Haptotaxis. Methods in Enzymology 570, 567–581 (2016). 26921962 10.1016/bs.mie.2015.11.004

