# Supplementary material for: A microfluidic device for measuring cell migration towards substrate-bound and soluble chemokine gradients
Source: Sci Rep. 2016 Nov 7;6:36440. doi: 10.1038/srep36440 (PMC5098208; doi:10.1038/srep36440)
Supplement: Supplementary Information [file srep36440-s1.pdf]

A microfluidic device to measure cell migration towards substrate-bound and soluble chemokine gradients

Jan Schwarz[1], Veronika Bierbaum[1], Jack Merrin[1], Tino Frank[2], Robert Hauschild[1], , Tobias Bollenbach[1], Savaş Tay[2], Michael Sixt[1], Matthias Mehling[1,2,3]

[1]*Institute of Science and Technology Austria (IST Austria), Klosterneuburg, Austria*

[2]*Department of Biosystems Science and Engineering, ETH Zürich, Basel, Switzerland*

[3]*Department of Biomedicine and Neurology Department, University Hospital Basel, Basel, Switzerland*

correspondence:

Matthias Mehling, Department of Biomedicine, *University Hospital Basel*, Hebelstrasse 20, 4031 Basel, Switzerland; phone: +41(0)61 265 25 25, E-mail: matthias.mehling@unibas.ch;

or Michael Sixt, Institute of Science and Technology Austria, Am Campus 1, Klosterneuburg, Austria; phone: +43 (0)2243 9000-3801, E-mail: michael.sixt@ist.ac.at

Supplemental information

Supplemental movie S1: Representative movie of DCs migrating in the microfluidic migration chamber. Left panel: DCs migrating on fibronectin. Middle panel: Haptokinetic migration of DCs on fibronectin functionalized homogenously with CCL21 24-98 bio. Right panel: Haptotactic migration of DCs on fibronectin functionalized with an exponential gradient of CCL21 24-98 bio. Scale bars represent 50 µm.

Supplemental movie S2: Representative movie of DCs following a gradient of CCL19 in the microfluidic migration chamber. The three different concentration regimes are indicated as black boxes ($CCL19_{high}$, $CCL19_{medium}$ and $CCL19_{low}$). CCL19/TAMRA dextran 10 kDa gradient in red. Scale bar represents 50 µm.

Supplemental movie S3: Representative movie of DCs migrating on printed CCL21 24-98 bio gradients (red). Cells are challenged with a soluble CCL19/FITC dextran 10 kDa gradient in green. Tracked areas lie within the three different concentration regimes ($CCL19_{high}$, $CCL19_{medium}$ and $CCL19_{low}$) and are indicated as black boxes. Scale bar represents 50 µm.

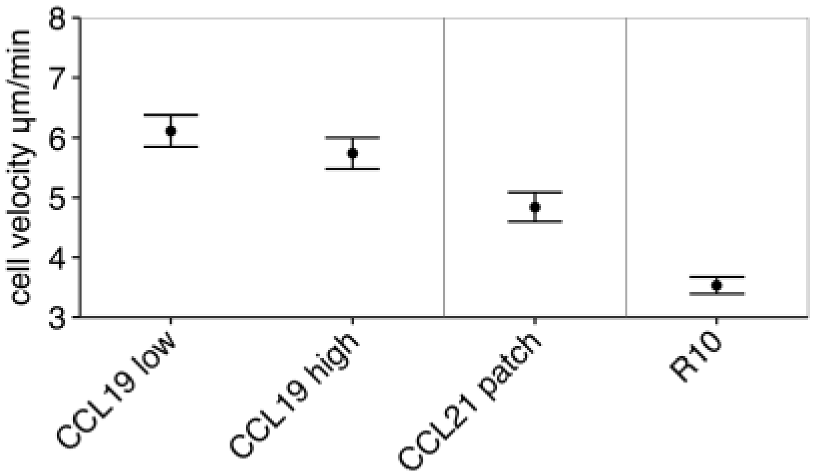

Supplemental figure S4: Velocities of DCs for different conditions: cells migrate with increased velocity in presence of both CCL19 and CCL21 in comparison to medium only (R10). Velocities are increased even if the cells do not move directionally, as is the case at low concentrations of soluble CCL19 and on the patch of CCL21.

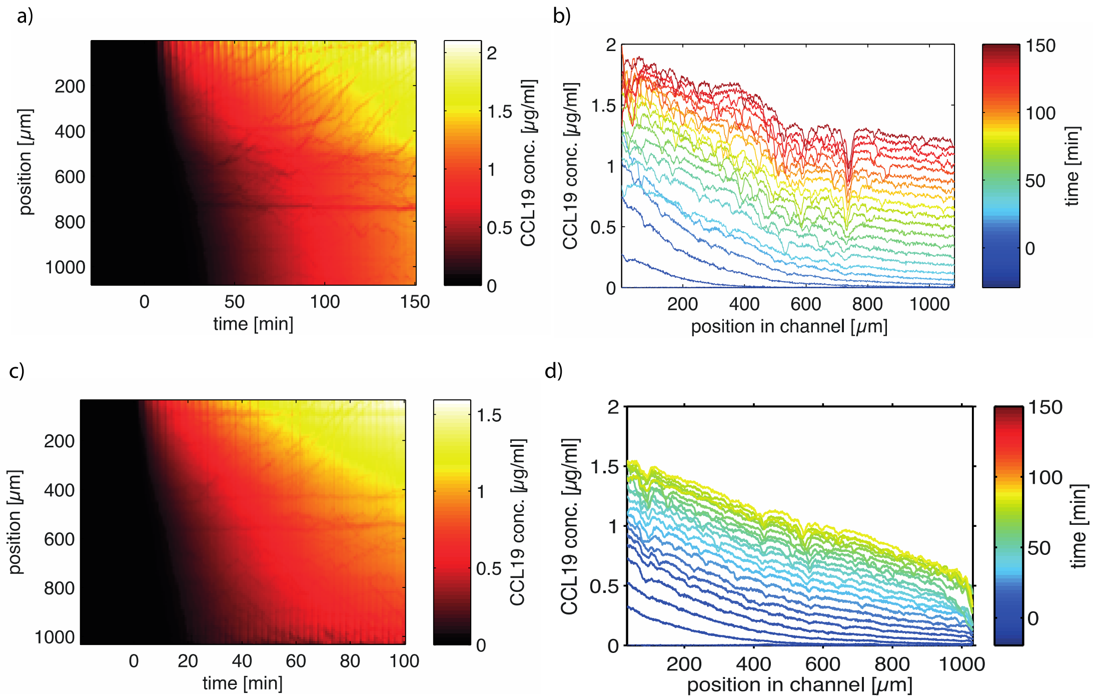

Supplemental figure S5: Variation of CCL19 concentration in the experiments shown in Figure 4 (a, b) and 5 (c, d) of the main text as a function of time. Left panels (a, c): CCL19 concentration indicated by a heat map across the chamber, where dark colours indicate low and light colours indicate high concentrations. As the microfluidic device is operated, CCL19 enters at the top of the chamber (position 0), and is flushed away at the chamber bottom, by continuous opening/closing of the valves of the channel. Time t=0 corresponds to the first entry of chemokine into the chamber, and the profile of CCL19 builds up over time. The traces are due to cells moving in the chamber. Right panels (b, d) Concentration of CCL19 as a function of position in the chamber for different times. Blue colours are for short, and red colours are for long times, with lines separated by 7.5 minutes (b) and 5 minutes (d). Fluctuations in the profile are from 'shadows' of migrating cells.

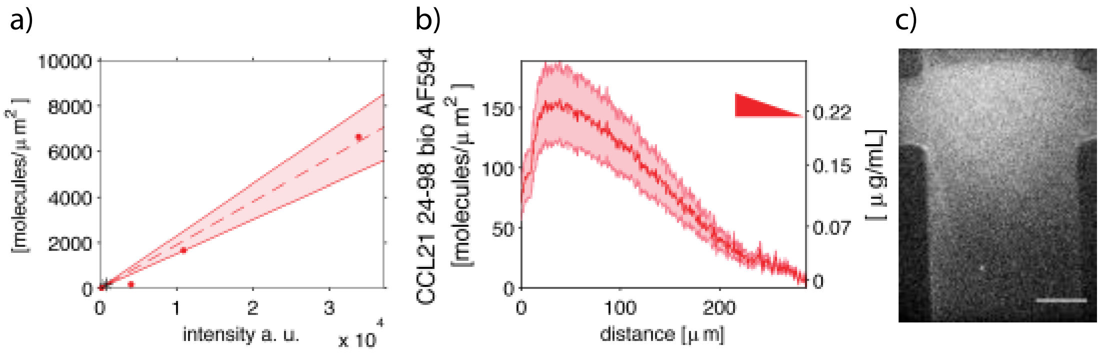

Supplemental figure S6: Calibration curve and quantification of immobilized CCL21 profile. (a) Calibration curve. Soluble CCL21 of varying concentrations (0.25, 1.0, 2.5 and 10 μg/mL) immersed in the chamber, rescaled from observed area to molecules per μm^2, plotted as a function of fluorescence intensity in a given area. The fit function for conversion reads (molecules/μm^2)=(0.190 ± 0.039)*intensity. The shading shows upper and lower bounds of the uncertainty of calibration. The black star indicates the maximal intensity of the immobilized CCL21. (b) Concentration of immobilized CCL21, averaged over the width of the profile, with shades indicating the uncertainty in calibration. (c) Snapshot of immobilized CCL21 in the chamber. The scale bar is 50 μm.
